# Metabotyping Patients’ Journeys Reveals Early Predisposition to Lung Injury after Cardiac Surgery

**DOI:** 10.1038/srep40275

**Published:** 2017-01-11

**Authors:** Raluca Georgiana Maltesen, Bodil Steen Rasmussen, Shona Pedersen, Munsoor Ali Hanifa, Sergey Kucheryavskiy, Søren Risom Kristensen, Reinhard Wimmer

**Affiliations:** 1 Department of Anaesthesia and Intensive Care, Aalborg University Hospital, Denmark; 2 Department of Chemistry and Bioscience, Aalborg University, Denmark; 3 Department of Clinical Medicine, Faculty of Medicine, Aalborg University, Denmark; 4 Department of Clinical Biochemistry, Aalborg University Hospital, Denmark

## Abstract

Cardiovascular disease is the leading cause of death worldwide and patients with severe symptoms undergo cardiac surgery. Even after uncomplicated surgeries, some patients experience postoperative complications such as lung injury. We hypothesized that the procedure elicits metabolic activity that can be related to the disease progression, which is commonly observed two-three days postoperatively. More than 700 blood samples were collected from 50 patients at nine time points pre-, intra-, and postoperatively. Dramatic metabolite shifts were observed during and immediately after the intervention. Prolonged surgical stress was linked to an augmented anaerobic environment. Time series analysis showed shifts in purine-, nicotinic acid-, tyrosine-, hyaluronic acid-, ketone-, fatty acid, and lipid metabolism. A characteristic ‘metabolic biosignature’ was identified correlating with the risk of developing postoperative complications two days before the first clinical signs of lung injury. Hence, this study demonstrates the link between intra- and postoperative time-dependent metabolite changes and later postoperative outcome. In addition, the results indicate that metabotyping patients’ journeys early, during or just after the end of surgery, may have potential impact in hospitals for the early diagnosis of postoperative lung injury, and for the monitoring of therapeutics targeting disease progression.

Cardiovascular disease is society’s number one health problem, being the leading cause of death in many countries^1^,^2^. Annually worldwide, nearly one million patients undergo coronary artery bypass grafting (CABG) surgery with the use of cardiopulmonary bypass (CPB)^3^. Even after uncomplicated procedures, some patients experience a systemic inflammatory response and ischemia-reperfusion lung injury. Lung injury consists of a series of pathophysiological changes, including pulmonary oedema, decreased lung compliance, disturbance in the ventilation-perfusion ratio, and increased pulmonary vascular resistance. This leads to postoperative hypoxaemia and, in the most severe cases, to acute respiratory distress syndrome (ARDS)^4^,^5^,^6^. Other causes of postoperative hypoxaemia are atelectasis in the immediate postoperative period^7^ and fluid accumulation in the lung^8^. Studies of the gas exchange parameters on the third postoperative day after CABG show a decrease in shunt fraction, an increase in ventilation-perfusion mismatch due to less atelectasis, and an increase in fluid accumulation and lung injury^9^,^10^.

Since postoperative impairments in lung function extends hospitalization and increases various complications, as well as mortality^6^,^11^, early identification of at-risk patients would be advantageous. Determining the progression into hypoxaemia is challenging, as no early diagnostic test exists^12^. Early measurements of the partial pressure of arterial oxygen (PaO_2_) -used to assess the degree of acute lung injury^13^,^14^ - have shown poor predictive value for later outcomes^15^. Because the nadir values of PaO_2_ appear on the second to third postoperative day^9^,^10^, it is difficult to predict which patients will develop lung injury at an early stage^16^.

Currently, there are no proven treatment options^11^,^17^ and no molecular-driven interventions^18^,^19^,^20^ to prevent disease progression. Therefore, an understanding of the risk factors and molecular mechanisms may help preventing hypoxaemia^6^. A patient’s medical history before cardiac surgery (e.g. age, health state, and smoking habits)^4^,^21^,^22^, previous cardiac surgery^23^, the surgical procedure itself (general anaesthesia, sternotomy, atelectasis, and the use of CPB)^4^,^24^,^25^,^26^,^27^, and blood transfusion^23^ are well known risk factors of postoperative lung injury. Polymorphisms in the pro-inflammatory interleukin-encoding genes of IL-6 and IL-18 have been shown to predispose patients to CPB-induced acute lung injury^28^,^29^. In addition, increased circulating free fatty acids two hours after CABG have been identified as being early signs of postoperative hypoxaemia^6^. In line with these findings, we have recently shown that it was possible to predict PaO_2_ measured on the third day postoperatively from a blood sample collected on the first postoperative morning^30^. A pattern of disturbed metabolism was observed, of which changes in ketones, amino acids, and lipid metabolism were dominant.

While these molecular mechanisms are crucial for the early prognostication of at-risk patients, there is still need for a better understanding of the molecular reasons as to why certain patients develop lung injury, while others do not. Hence, the aim of this study was to investigate the time course of metabolic events, from the start of the operation to the development of hypoxaemia measured on the third postoperative day. Furthermore, we investigated whether it was possible to find a specific ‘metabolic biosignature’ that correlated with the risk of developing postoperative lung injury defined by hypoxaemia.

We adopted a metabonomics approach, since it aims to find insights into the actual metabolic phenotype (metabotype^31^) of diseases, and the causes of their progression^32^. Because of serial sampling, each patient served as its own control. This allowed us to create an individual ‘metabolite journal’ and to follow each metabolite profile from prior to surgery until the day of diagnosis.

## Results

### Patient characteristics

Patient characteristics, surgical variables, and the PaO_2_ levels are given in Table 1.

**Figure 1:**
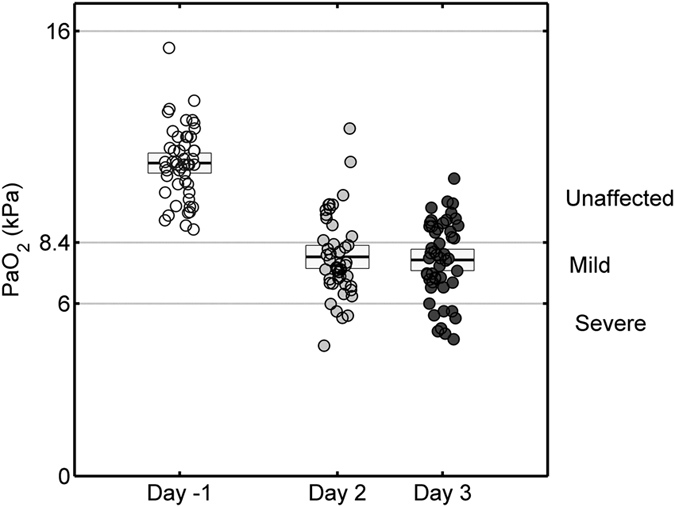
Partial pressure of oxygen (PaO_2_) measured in arterial blood on 50 fasting patients the day before surgery (Day-1), and the second and third day postoperatively, to assess the degree of hypoxaemia. Arterial PaO_2_ levels showed an averaged 29% decrease on the second (48 hours) (analysis of variance, ANOVA, p < 0.0001) and a 31% (p < 0.0001) decrease 72 hours postoperatively compared to their baseline levels. Based on PaO_2_ values obtained on the third postoperative day, patients were divided into hypoxaemic patients (‘mild’ with 8.4 > PaO_2_ ≥ 6.3 kPa, ‘severe’ with PaO_2_ < 6.3 kPa) and unaffected patients with PaO_2_ ≥ 8.4 kPa.

Decreased arterial PaO_2_ levels were observed on the second and third postoperative days compared to the day preceding surgery (Fig. 1). The diagnosis of hypoxaemia was based on the PaO_2_ values measured 48 and 72 hours after weaning from CPB during spontaneous breathing with a fraction of inspired oxygen (FiO_2_) and defined as PaO_2_ < 8.4 kPa or PaO_2_/FiO_2_ < 40 kPa. Forty eight percent of patients suffered hypoxaemia on the second, and 64% on the third postoperative day. Oxygenation worsened in some patients, whilst it improved in others, between the second and third days. Hence, patients’ outcome was defined based on the third day’s oxygenation values. Eighteen patients did not develop hypoxaemia (‘unaffected’), while thirty-two patients experienced hypoxaemia, of which nine suffered severe oxygen impairments (PaO_2_ ≤ 6.3 kPa or PaO_2_/FiO_2_ ≤ 30 kPa).

There was no significant difference between the groups in age, body mass index, and smoking habits, however, gender was slightly different (p = 0.01). Longer surgical procedure was observed in the hypoxaemic groups; however, the difference was statistically insignificant. In addition, no difference in the perioperative fluid balance (gain), administrated cardioplegia solution, and medication was observed, and no patient received glucose infusion during the surgical procedure. There was no difference in time of mechanical ventilation and length of stay in the cardiothoracic intensive care unit; however, two patients were readmitted for non-invasive ventilation due to severe hypoxemia. Weight gain and the inflammatory parameters were significantly higher in the severe hypoxaemia group. Also, a tendency towards longer length of stay at the hospital was observed in these patients. There was no difference in the incidence of postoperative atrial fibrillation.

### Metabotyping the patient journey

For this study 738 blood samples were collected at nine different time points: the day before surgery (samples number, n = 50), intraoperatively (after sternotomy, but before CPB, n = 100; immediately after weaning from CPB or ‘0 hour’, n = 100), and at 2 (n = 100), 4 (n = 100), 8 (n = 100), 20 (n = 94), 48 (n = 47), and 72 hours (n = 47) post-CPB. Samples obtained from arterial blood the day before, 48 and 72 hours postoperatively were of plasma specimen, while samples obtained simultaneously from the catheters inserted in the pulmonary artery (PA) and the left atrium (LA) intraoperatively, and the following 20 hours post-CPB were of serum specimen. PA and LA samples were obtained to investigate possible differences between the systemic and pulmonary microvasculature, respectively.

**Figure 2:**
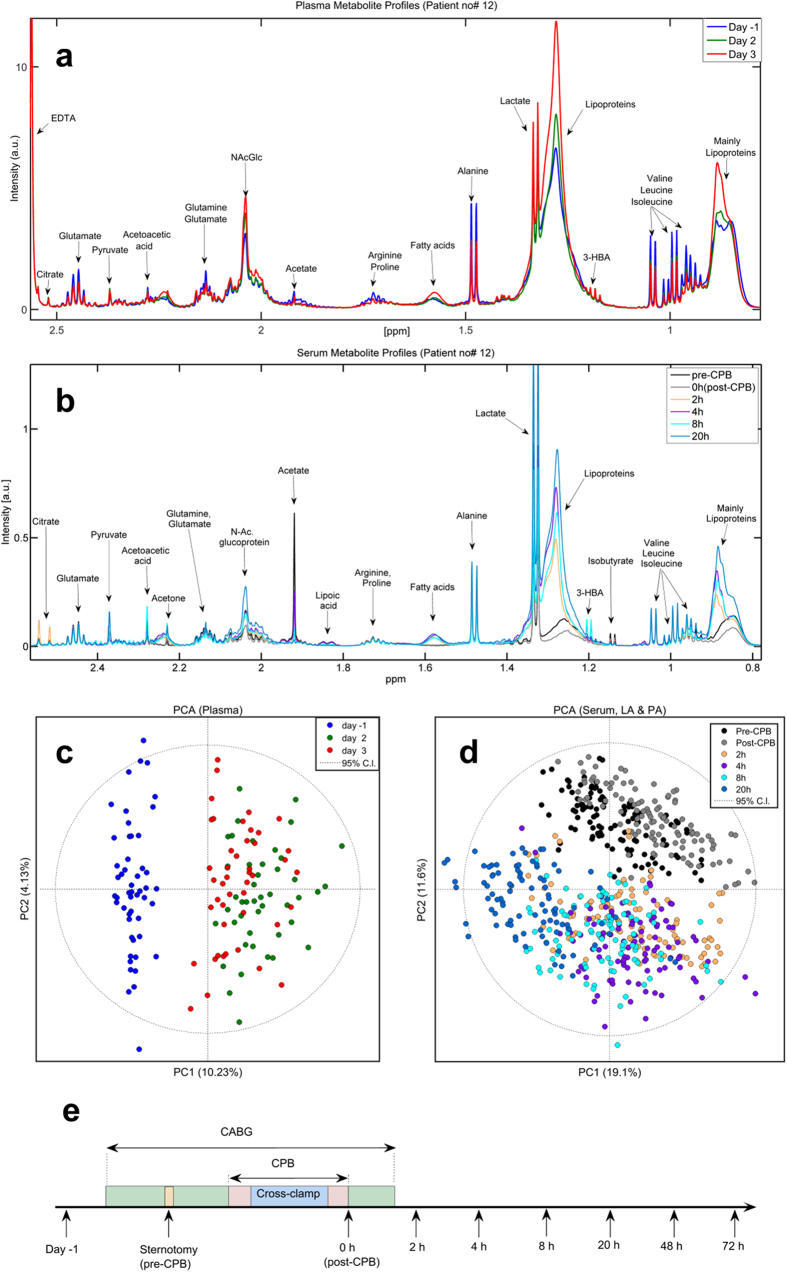
Metabotyping patients’ journeys. (**a**) Representative ^1^H-NMR Carr-Purcell-Meiboom-Gill (CPMG) spectra representing the metabolic profile of an arbitrary chosen ‘unaffected’ patient showing plasma collected the second (green) and third postoperative day (red) compared to the day before surgery (blue). (**b**) Metabolite changes observed in serum samples collected after sternotomy but before cardiopulmonary bypass (CPB), just after weaning from CPB (0 hour), and 2, 4, 8, and 20 hours postoperatively. (**c**) Principal component analysis (PCA) scores plot of 144 plasma samples collected the day before surgery, and the second and third day postoperatively. (**d**) PCA performed on 594 serum samples collected at six different time points intraoperatively and the following 20 hours post-CPB from both the left atrium and pulmonary artery. In addition, a time line explaining the surgical events and the samples collected for this study is provided. Samples were collected the day before surgery, after sternotomy, at the end of CPB (0 hour), and 2, 4, 8, 20, 48, and 72 hours post-CPB.

Each patient’s metabolic journey from prior to surgery, during the procedure, and the three postoperative days was metabotyped. Decreases in the levels of several amino acids and increases in the levels of ketones, fatty acids, and lipids were observed in both plasma (Fig. 2a) and serum samples (Fig. 2b). These changes were consistently seen across all patients.

To systematically analyse metabolome response to surgery, principal component analysis (PCA) was performed on plasma (Fig. 2c) and serum (Fig. 2d) samples. PCA recognized patients according to the time at which their samples had been collected, and clustered them accordingly. Samples collected during surgery (pre-CPB, black; 0 hour post-CPB, grey) clustered along the second principal component (PC2); while postoperative samples migrated from 2 (orange) to 4 (purple), 8 (light blue) and to 20 hours (blue green) along PC1. Although the 20 hours sample scores approached perioperative samples, they did not overlap. By 48 hours samples were still not co-mapping preoperative values; while by 72 hours sample scores had moved towards baseline values, suggesting that the metabolome had almost recovered from surgery.

### The surgical procedure affects the metabolome

**Figure 3:**
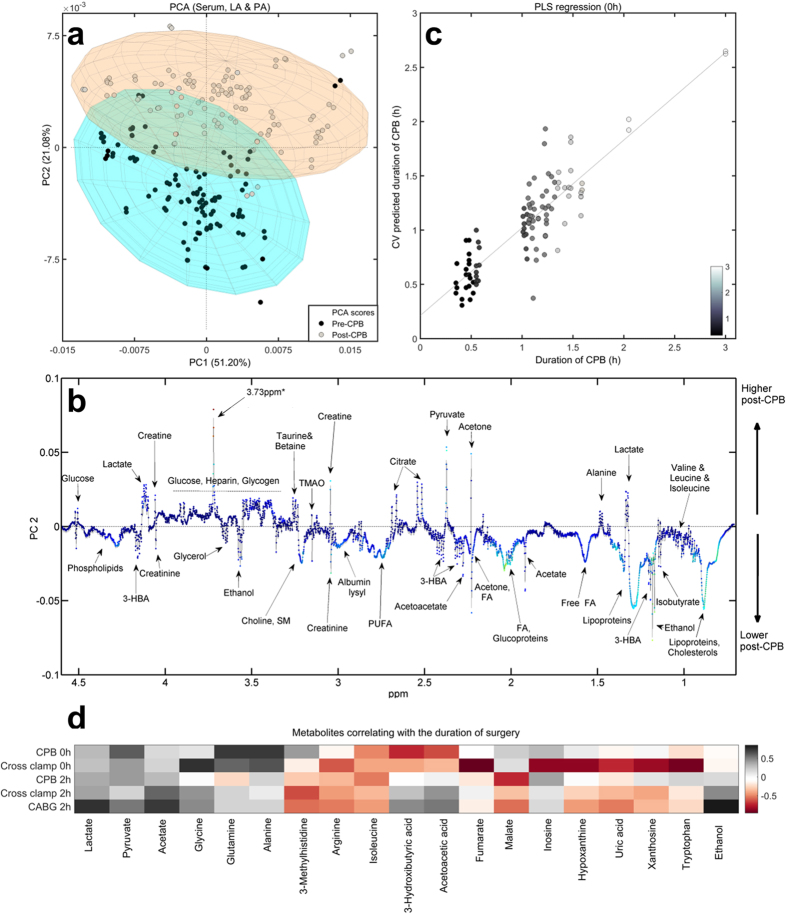
Metabolome changes as a consequence of the surgical procedure. (**a**) Principal component analysis (PCA) performed on serum samples collected intraoperatively (‘pre-CPB’: after sternotomy, but before CPB; ‘post-CPB’: immediately after CPB, at 0 hour). (**b**) The corresponding loadings plot shows metabolite composition on PC2. (**c**) Partial least-square (PLS) regression analysis performed on serum NMR spectra from samples collected immediately after weaning from CPB showed cross-validated (CV) associations with the actual duration of CPB. For validation purpose a ten-fold Venetian-Blinds CV was employed. (**d**) Metabolites found to correlate with the duration of CPB, cross-clamp, and CABG, and their time-line trends until 2 hours post-CPB (black: positive correlation; white: no significant correlation; red: negative correlation). Abbreviations: 3-HBA, 3-hyroxybutyric acid; FA, fatty acids; PUFA, polyunsaturated fatty acids; TMAO, trimethylamine-N-oxide; ppm, parts per million; 3.73 ppm*, metabolite putatively identified as poly ethylene glycol (a widely used excipient for drug formulation).

To investigate how the surgical procedure influenced the human metabolome, PCA was performed on 200 PA and LA serum samples collected intraoperatively. Samples clustered according to the time of collection (Fig. 3a), suggesting that CPB induced changes in metabolite levels. Postoperatively, the levels of circulating glucose, pyruvate, alanine, lactate, citrate, and creatine increased, while the levels of phospholipids, free fatty acids (free FA), polyunsaturated fatty acids (PUFA), choline-containing compounds, lipoproteins, glycerol, ketones (3-hydroxybutyric acid, acetate, acetoacetic acid), and several other amino acids decreased (Fig. 3b, Table 2).

Similar changes were observed in both PA and LA samples, with a few exceptions (Table 2). Lactate increased by 33% and pyruvate by 61% in PA samples post-CPB; in comparison, their levels were further elevated in the LA samples. The purine metabolites including inosine, hypoxanthine, uric acid, and xanthine decreased, especially in the PA samples, suggesting their release from the lungs. In contrast, citrate and N-Ac-glycoprotein fragment levels were more elevated in the PA samples, suggesting their consumption by the lungs. The levels of ketones, including 3-hydroxybutyric acid (3-HBA), acetoacetic acid and acetate, and the branched-chain amino acids (BCAA) leucine and isoleucine had similar increases in LA and PA samples. Most lipids and fatty acids showed similar decreases in both PA and LA, except for diacylglycerophosphocholine (DAGPL), which was lower in LA samples, suggesting its utilization by the lungs.

We also evaluated how prolonged surgical procedures affected the metabolome. Partial least-square (PLS) regression analysis performed on samples collected at 0 hour against the duration of CPB, gave a moderate ten-fold Venetian-Blinds cross-validated (CV) coefficient of determination (R_cv_ = 0.76) (Fig. 3c), suggesting that a longer time on bypass stressed the metabolome. Longer aortic cross-clamp (ischemic) and CABG times were also found to influence the metabolome (Supplementary data Table S1).

Lactate, pyruvate, and acetate levels positively correlated with the duration of ischemia and CPB at 0 hour, and with the duration of CABG at 2 hours, suggesting their increased production with the length of surgery (Fig. 3d). Glycine, alanine, and glutamine concentrations correlated with the duration of CPB and cross-clamp exclusively at 0 hour. Arginine, isoleucine, and 3-methylhistidine negatively correlated with cross-clamp and CABG time, indicating their utilization with prolonged surgical stress. Acetoacetate and 3-HBA negatively correlated with the duration of CPB and cross-clamp at 0 hour, and positively correlated with the duration of CABG at 2 hours post-CPB. The tricarboxylic (TCA) cycle intermediates fumarate and malate, and the purine metabolites, inversely correlated with the surgical time. Finally, ethanol correlated with the duration of CABG, suggesting increased antiseptic use with prolonged surgical time.

The associations did not persist for more than 2–4 hours, suggesting a normalization of the metabolome after longer procedures.

### The post-CPB period

**Figure 4:**
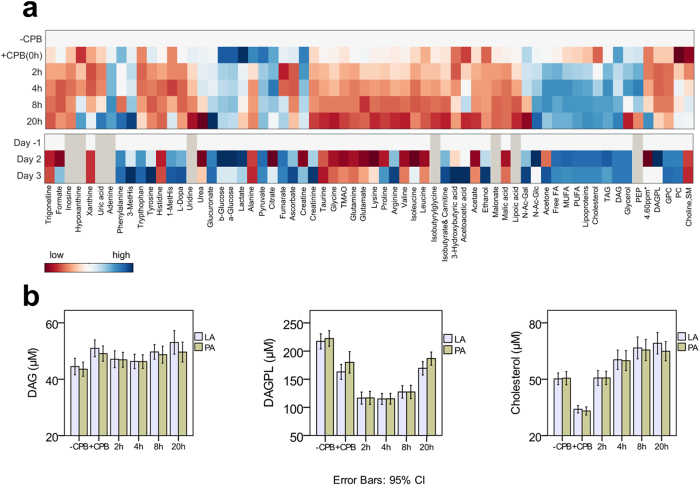
Patients’ metabolic journeys. (**a**) Heat map representation of time-dependent metabolic changes occurring within the nine different time points (the day before surgery, ‘Day-1’; before cardiopulmonary bypass, ‘pre-CPB’, immediately after weaning from CPB ‘0 hour’, and 2, 4, 8, 20, 48, and 72 hours post-CPB. The colours are based on the mean changes (in percentages) calculated as (post-CPB − pre-CPB)/pre-CPB*100. Grey colour represents metabolites that could not be detected in plasma samples. (**b**) Time line for selected metabolites measured in samples collected from the left atrium (LA) and pulmonary artery (PA). Means and standard deviations (error bars represent 95% confidence intervals) are provided. Abbreviations: 1, 3-MetHis., 1- and 3- methylhistidine; TMAO, trimethylamine-N-oxide; N-Ac-Gal, N-acetyl-galactosamine; N-Ac-Glc, N-acetyl-glucosamine; FA, fatty acids; MUFA, monounsaturated fatty acids; PUFA, polyunsaturated fatty acids; DAG, diacylglycerol; PEP, phosphoenolpyruvate; ppm, parts per million; 4.60 ppm*, unassigned metabolite; DAGPL, diacylglycerophosphocholine; PC, phosphatidylcholine; GPC, glycerophosphocholine; SM, sphingomyelin.

Postoperatively, several metabolites recovered their pre-CPB levels within the first 2–4 hours; however, most metabolites continued changing until 20 hours. In fact, their levels had not returned to baseline at 48 hours (Fig. 4a). Glycolytic and TCA cycle metabolites were mostly elevated in the early postoperative period, but returned towards baseline levels in the following 20–48 hours. The levels of purine metabolites, nicotinic acid metabolites (trigonelline, tryptophan), tyrosine metabolites (tyrosine, L-dopa), histidine, and uridine were low post-CPB, and few of them reached their pre-CPB levels within 8–20 hours. In contrast, adenine, phenylalanine, 3-methylhistidine, glucuronate, and N-Acetyl glucosamine (N-Ac-Glc) were increased postoperatively, and their levels, except N-Ac-Glc, continued to rise at 48 to 72 hours. Lysine, BCAA, glutamine, glutamate, glycine, taurine, and trimethylamine‐N‐oxide (TMAO) levels were reduced even after 72 hours.

Most fatty acid and lipid concentrations decreased immediately after surgery; however, steep increases occurred after 2 hours post-CPB. Free fatty acids (free FA) levels decreased by 30% at the end of CPB, and increased to 2-fold pre-CPB levels after 2 hours, where the sedation was changed from inhalation anaesthesia to intravenous propofol administration. Increases in the levels of these metabolites were observed even after propofol administration was stopped, at 4 hours postoperatively. The same trends were observed for polyunsaturated fatty acids (PUFA), di- and triacylglycerol (DAG, TAG), and lipoproteins. On the other hand, lipoic acid concentrations decreased continuously after CPB, to 70% at 20 hours post-CPB, and its signals vanished at 48 hours. Choline-containing compounds including DAGPL, glycerophosphocholine (GPC), and phosphatidylcholine (PC) were all found to be decreased postoperatively.

Similar trends were observed in LA and PA samples (Supplementary Table S2). While the levels of some metabolites were different at baseline (pre-CPB), their levels were similar in the following 4–8 hours, and returned to the baseline differences afterwards (Fig. 4b). Phenylalanine, nicotinic acid metabolites, most purine metabolites, tyrosine metabolites, histidine, 1-methyl-histidine, uridine, glucuronate, lactate, pyruvate, acetone, cholesterol, and DAG were all elevated in LA samples in at least one time point, indicating their release from the lungs. Tryptophan, hypoxanthine, ascorbate, and DAGPL were elevated in PA samples in at least one time point, indicating their utilization by the lungs.

### Phenotyping patients’ journeys revealed predisposition to lung injury defined by hypoxaemia

Due to the changes observed, we explored the potential diagnostic value of the metabolome for the early detection of hypoxaemia.

**Figure 5:**
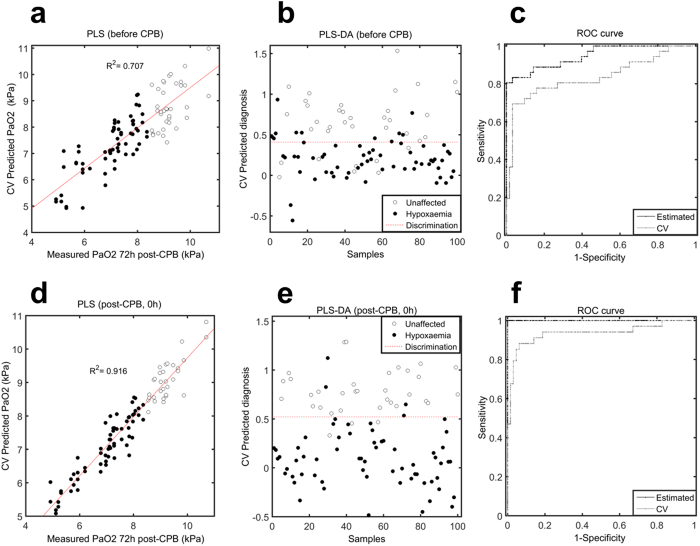
Early predispositions to lung injury defined by hypoxaemia. (**a**) Partial least-square (PLS) regression plot shows cross-validated association between the metabolome measured after sternotomy, but before CPB, with arterial PaO_2_ values measured 72 hours postoperatively. A ten-fold Venetian-Blinds CV was used for validation purpose. (**b**) Partial least-square discriminant analysis (PLS-DA) prediction scores plot of the cross-validated model, which discriminates between patients who will subsequently develop hypoxaemia (black) and patients who will not be affected by hypoxaemia (white), using serum samples taken after sternotomy, but before CPB. (**c**) The corresponding receiver operating characteristic (ROC) curve showing the predictive capacity of the model, with both calibrated (Estimated, black line) and cross-validated (CV, grey line) results, for samples collected after sternotomy. (**d**) PLS regression plot showing cross-validated correlation between the metabolome measured after weaning from CPB and PaO_2_ values. (**e**) PLS-DA prediction scores plot of the model discriminating hypoxaemic (black) from unaffected patients (white), using serum samples taken immediately after weaning from CPB. (**f**) The corresponding ROC curve.

PLS regression analysis, carried out to map metabolome onto arterial PaO_2_ values obtained 72 hours postoperatively, showed moderate ten-fold Venetian-Blinds CV association (R^2^_cv_ = 0.71) already with samples taken after sternotomy but before CPB (Fig. 5a, Supplementary data Table S3). The validity of using the metabolome for accurately distinguishing hypoxaemic from unaffected patients was investigated by building and cross-validating a discrimination model based on these samples (Fig. 5b). PLS-DA revealed 77.8% sensitivity and 84.2% specificity towards differentiating later outcomes (Fig. 5c). Model robustness was assessed by randomly permuting each patient label 500 times and performing the modelling. The real model outperformed the permuted models (p = 0.004).

A larger association, sensitivity, and specificity were observed when analysing 0 hour samples (Fig. 5d–f), indicating that the CPB and cross-clamp procedures played significant roles in the development of lung injury. Modelling performed on samples collected at later time points confirmed these results (Supplementary Table S3).

**Figure 6:**
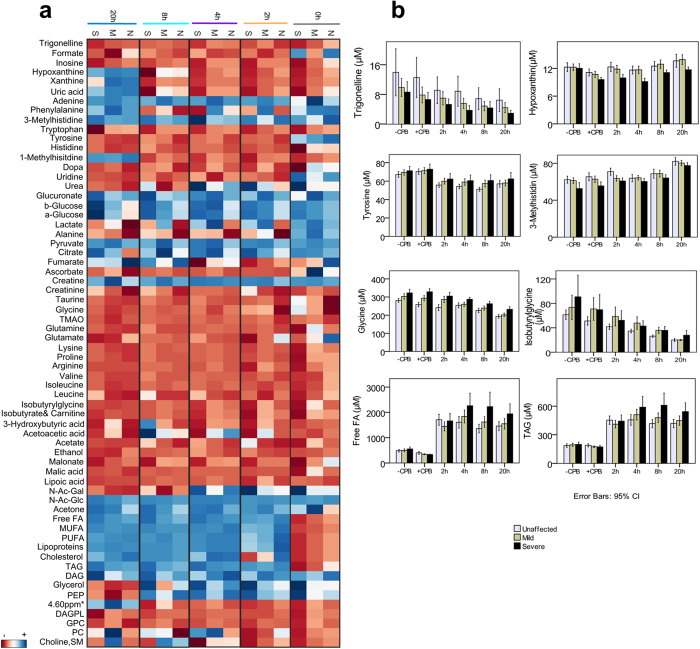
Metabolic signature of lung injury. (**a**) Heat map representation of the mean percent changes in the unaffected (‘N’), mildly affected (‘M’), and severely affected (‘S’) by hypoxaemia patients. Means percent changes were calculated as (post-CPB − pre-CPB)/pre-CPB*100. (**b**) Selected metabolites showing time- and phenotype dependent changes Means and standard deviations (error bars represent 95% confidence intervals) are provided. Abbreviations: ‘−’, decreased levels; ‘+’, increased levels; TMAO, trimethylamine-N-oxide; N-Ac-Gal, N-acetyl-galactosamine; N-Ac-Glc, N-acetyl-glucosamine; FA, fatty acids; MUFA, monounsaturated fatty acids; PUFA, polyunsaturated fatty acids; DAG, diacylglycerol; PEP, phosphoenolpyruvate; 4.60 ppm*, unassigned metabolite; DAGPL, diacylglycerophosphocholine; PC, phosphatidylcholine; GPC, glycerophosphocholine; SM, sphingomyelin.

The metabolites found to differentiate hypoxaemic from unaffected patients are summarized in Fig. 6a and Table 3. We divided hypoxaemic patients according to their PaO_2_ levels into mild (8.4 > PaO_2_ > 6.3 kPa) and severe group (PaO_2_ ≤ 6.3 kPa), to emphasize the level of impairment and the degree of later lung injury. The most discriminatory metabolites were involved in purine metabolism, nicotinic acid metabolism, methylhistidine metabolism, tyrosine metabolism, glycine and isobutyrylglycine metabolism, fatty acid and lipid metabolism, and N-Acetyl-glucosamine metabolism (Fig. 6b). When analysing samples taken just after sternotomy, purine metabolites, nicotinic acid metabolites, and 3-methylhistidine levels positively correlated with later PaO_2_ measurements; while tyrosine, L-dopa, glycine, and isobutyrylglycine levels inversely correlated to the degree of oxygenation. The patterns within most of these metabolites continued to be different between groups, suggesting their potential as early markers of postoperative lung injury (Table 3). Moreover, the levels of glycerol discriminated patients at time point 0 and 2 hours, with highest levels in the severe hypoxaemic group. The levels of free fatty acids and lipoproteins discriminated patients at time point 4 hours, and their levels were inversely correlated with PaO_2_. N-Acetyl glucosamine, pyruvate, alanine, glucuronate, histidine, glucose, lactate, creatine, creatinine, urea, arginine, valine, and isoleucine also differentiated patients in at least two of the measured time points postoperatively.

## Discussion

The aims of this study were to investigate the impact of cardiac surgery on the human metabolome and to highlight possible mechanisms involved in the progression of postoperative hypoxaemia. The study shows that the patients with the most severe hypoxaemia suffered a more pronounced inflammatory response, had more fluid accumulation, and a prolonged length of stay in hospital. The PaO_2_/FiO_2_ ratio at the time of weaning from mechanical ventilation was slightly lower in the severe hypoxaemic patients, and as such, atelectasis is probably part of the mechanism of pulmonary impairment followed by lung injury with capillary leakage and inflammation.

The design of the study allowed us to focus specifically on the immediate metabolic effects of the surgical procedure, and to determine whether a metabolite was released, consumed, or just transiently passed through the lungs. Analysing samples from nine time points allowed us to create a metabolic journal and follow each patient through recovery from surgical trauma or towards progression into lung injury. Since each patient was their own control, potential confounding factors arising from different study populations and surgical units were minimized.

There are several significant findings of the present study. Firstly, NMR spectroscopy detected immediate changes in the metabolite profiles as a consequence of cardiac surgery (Fig. 2). Secondly, prolonged surgical procedures impacted the metabolome (Fig. 3, Table 2). Thirdly, the intraoperative metabolome indicated a possible predisposition to lung injury (Fig. 5), allowing us to identify a ‘metabolic biosignature’ (Fig. 6, Table 3) which correlated with the risk of later hypoxaemia.

### The intraoperative period

During the surgical procedure, the beating of the heart and the pulmonary ventilation were stopped, the lungs were allowed to collapse, and the blood flow through the lungs was limited. For this period, CPB took over the functions of the heart and lungs, pumping oxygenated blood through the body. Since we collected blood after sternotomy, but before CPB, and right after weaning from CPB (0 hour), it was possible to explore the impact of CPB on the human metabolome. We observed dramatic and immediate shifts in the metabolome. PCA showed clear separation between pre- and post-CPB samples (Figs 2d and 3a,b), indicating a global metabolic change and a possible stress response to the procedure.

Because the surgical procedure was an important trigger for the later progression of hypoxaemia, we focus here on explaining the possible metabolic derangements found during the procedure.

It is worth mentioning that the changes observed post-CPB are a combination of reactions in the lungs and the cardioplegic heart just as cardioplegia fluid will flow into the blood when the heart starts beating. Our results include the inseparable sum of these effects, although ischemia of the lungs probably is the most important component, since cardioplegia is supposed to maintain the metabolism of the cardiac cells. Besides cardioplegia, the anaesthetics used and haemodilution may also contribute to the metabolic changes observed post-CPB. We have previously shown that plasma albumin decreased by approximately 65% at the end of CPB^33^, indicating that haemodilution has taken place. However, since albumin also diffuses into the extravascular space, it was difficult to fully correct for the haemodilution effect. In this study, if we examine the post- and pre-CPB concentrations of creatinine or urea, which are both distributed in most of the body fluid, their levels are seen to change to a minor degree (Supplementary Table S2). Since these metabolites may also be affected by trauma and kidney function, it is difficult to accurately correct for the surgical effects, and therefore, we have chosen to present the unadjusted metabolite levels. Finally, when trying to associate the results to biochemical reactions it must be emphasized that the ischemic and reperfusion reactions primarily take place intracellularly and we only see a mirror of this in blood samples. Our findings are consistent with a general reliance on the anaerobic metabolism of glucose to generate ATP; a switch from net lactate consumption to lactate release; a fall in ketone extraction; and a dysregulation of fatty acid oxidation. Previous smaller studies on surgical ischemia-reperfusion^34^,^35^,^36^ have reported similar responses to surgical trauma. In comparison to these studies, we have detected a wider range of metabolites, have analysed both the systemic and pulmonary responses to CPB, and have linked these changes to the progression to lung injury. Hence, this study gives us new insights into the metabolic responses to surgery.

As patients did not receive glucose during surgery, increased glucose levels could be due to decreased cellular uptake, a common signature of surgical trauma^24^,^34^,^35^. It is a common finding that during ischemia and hypoxia, intracellular lactate accumulates, pH decreases, and lactate is released into the circulation to avoid cellular swelling and cell death^34^,^35^. In this study we observed increased levels of serum lactate, possibly suggesting that cells experienced an ischemic environment, which was more severe in the pulmonary microcirculation and released to the blood stream during reperfusion. Simultaneously, increased pyruvate levels suggest that pyruvate was diverted away from the pyruvate-dehydrogenase reaction towards the anaerobic lactate-dehydrogenase reaction, and towards alanine formation. Citrate increased by 30% right after CPB, and since this metabolite was a component of the cardioplegia solution administered during surgical procedure, its increase may be partially explained by this factor. In addition, we observed a decrease in ketone levels by ~17% and in fatty acid and cholesterol levels by ~30%. Decreased ketone levels and inhibition of fatty acid beta-oxidation have previously been related to ischemia-reperfusion^34^,^37^, hence, our results may suggest a deficiency in fatty acid oxidation.

A previous study performed on ischemic and hypoxic hearts has shown a 50% reduction of phosphatidylcholine in the ischemic heart, and a 22% reduction in the hypoxic heart^38^. We found a 17% reduction in serum choline and PC; a 19% reduction of DAGPL in PA, and 25% in LA samples; and a 7% reduction in GPC. These findings suggest severe impairments in their biosynthesis during CPB. In addition, glycerol and DAG levels increased, especially in the LA samples after weaning from CPB. Their releases have previously been linked to ischemia-reperfusion injury^37^, and the results may therefore indicate potential cell membrane degradation and cellular injury during CPB.

At the end of CPB we also found impairments in the levels of several amino acids. One of the interesting findings was that the levels of all purine metabolites were decreased. These metabolites are by-products of adenosine triphosphate (ATP) degradation, and are known to accumulate during ischemia due to reduced oxygen and to decrease during reperfusion, when oxygen is reintroduced^39^. We did not sample during the ischemic and reperfusion periods, however, patients undergoing prolonged aortic cross-clamping showed significant decreases in the purine levels (Fig. 6a,b). Apart from purine metabolites, we also observed that prolonged surgical procedures produced more anaerobic glycolytic compounds, and reduced the levels of the essential amino acids arginine and isoleucine.

### The postoperative period

Reobtaining homeostatic metabolism is an energy demanding process involving many metabolites^37^. We observed complex changes in the postoperative period. The picture that emerges postoperatively is one of profound impairments in the levels of lipids and metabolic fuels, likely reflecting severe deficiencies in energy metabolites, unbalanced pyruvate metabolism, and deranged ketone-, nicotinamide-, purine-, and hyaluronic acid metabolism (Fig. 4a).

Some metabolites recovered pre-CPB levels by 2–4 hours postoperatively; however, most metabolites continued changing until 20–72 hours. We noticed that several metabolites were elevated in either LA or PA samples during surgery (Fig. 4b, Supplementary data Table S2); however, their levels were similar 2–4 hours postoperatively, and then diverged, indicating the start of a possible systemic and pulmonary normalization.

Several lipids and fatty acids, which were reduced at the end of CPB, increased steeply in the postoperative phase, even several hours after propofol administration was stopped. The depletion may be explained by a combination of impaired biosynthesis, hemodilution occurring during CPB, and possible entrapment by the oxygenator filters; while the steep increases postoperatively may partially indicate the effect of propofol, but also possible lipolysis and cell membrane damage. Due to the combination of these effects, their reduction in concentration at the end of CPB may have been overestimated, while their increase post-CPB may have been underestimated.

Glucuronate, formed by glucose oxidation, was found to increase up to 72 hours postoperatively. Glucuronate is involved in the degradation and elimination of xenobiotics^40^ and in the synthesis of hyaluronic acid, along with N-acetyl-glucosamine^41^, which has been previously found to be involved in postoperative inflammation^36^, pulmonary diseases, and lung homeostasis^42^. N-acetyl-glucosamine positively correlated with glucuronate, and since their levels were slightly elevated in LA samples, it may indicate a possible hydrolysis of hyaluronic acid within the lungs. In addition, several amino acids changed in the postoperative period, probably reflecting impaired protein synthesis, enhanced proteolysis, or decomposition of proteins from skeletal muscles, which are all commonly observed after surgical trauma^43^. Changes may also be a result of various anti-inflammatory and volume regulating functions of specific amino acids as previously described^30^. It is worth mentioning that on the second and third postoperative day patients received nutritional support, and hence, some of the changes observed at a later time point may have been affected by nutrition.

### The link between the metabolome and the progression of lung injury

Metabonomics has previously been applied to patients undergoing major surgery^43^,^44^,^45^, and the approach has shown potential in predicting: systemic inflammatory responses and multi-organ dysfunction syndromes^43^, graft failure after kidney transplant^45^, and drug-toxicity after liver transplantation^44^. As with these studies, we showed that metabonomics has potential in predicting the development of postoperative lung injury already during the surgical procedure.

Remarkably, PLS regression analysis performed on samples obtained after sternotomy, but before CPB, showed a moderate CV association (R^2^_CV_ = 0.71) between the metabolome and arterial PaO_2_ values measured 72 hours postoperatively (Fig. 5a), indicating that even the initial procedure of sternotomy affected the metabolome in these patients. In accordance with this, Cristescu S.M. and co-workers have previously demonstrated that a marked lipid peroxidation (i.e. oxidative stress) was induced during sternotomy using diathermy^46^. In addition to this finding, our data revealed that sternotomy affected the levels of antioxidants (inosine, hypoxanthine) and metabolites involved in oxidative stress (trigonelline^47^), cell-volume regulation (glycine^48^,^49^), and tissue damage (3-methylhistidine^50^) (Fig. 6). Since these metabolites were also found to correlate with later oxygen levels, it might indicate their possible contribution to the postoperative hypoxaemia. While these findings have not been reported elsewhere, further studies are needed to confirm our results.

Besides sternotomy, we also found that the CPB and aortic cross-clamp periods significantly contributed to the development of later hypoxaemia (R^2^_CV_ = 0.92, Fig. 5b). Moreover, it was found that events occurring within the first day postoperatively (e.g. chest tube drainage, blood transfusion, and duration of ventilatory support) did not contribute significantly to patients’ outcomes, since the associations between the postoperative metabolome to the later PaO_2_ values were not markedly different to that observed after weaning from CPB (Supplementary Table S3). Hence, our results suggest that the surgical procedure is the main triggering factor of the progression to lung injury. The metabolites found to be affected by prolonged CPB and cross-clamp time were analysed for possible association to hypoxaemia. At 0 hours, pyruvate and alanine levels, which correlated with CPB and cross-clamp time, were also found to show slight association with later outcomes (Fig. 6b), indicating their links with surgical trauma and the development of postoperative lung injury. Glycine, found to correlate with the duration of aortic cross-clamp, had already increased in patients developing hypoxaemia after sternotomy, and continued to be discriminative of later outcomes even at 20 hours postoperatively. Hence, glycine may be a good biomarker candidate of postoperative lung injury. In addition, ketone metabolites inversely correlated with the length of CPB and the levels were lower in hypoxaemic patients. Finally, purine metabolites were negatively correlated to the length of aortic cross-clamp, and decreased in patients developing hypoxaemia, indicating impairments in their syntheses.

Several metabolites were found to be different between the groups, regardless of time on bypass. MUFA, PUFA, lipoproteins, and cholesterol showed discriminative value after weaning from CPB. Increased circulating free fatty acids have recently been reported as possible predictors of hypoxaemia at 2 hours post-CABG (r = −0.367, p < 0.001)^6^. We also report these changes straight after weaning from CPB; however, the changes become more significant 2–4 hours post-CPB, confirming previous findings. N-Ac-glucosamine levels increased postoperatively in all patients, and its levels at 20 hours were positively associated with the development of severe hypoxaemia. Nicotinic acid metabolites significantly decreased in hypoxaemic patients. Tyrosine metabolism (tyrosine, L-dopa) was decreased in all patients, but less decreased in hypoxaemic than in unaffected patients. Tyrosine metabolism has previously been related to ischemia-reperfusion injury and tissue damage^50^, and hence, the relatively elevated levels in hypoxaemic patients may indicate more ischemia-reperfusion injury and more tissue damage in these patients. Finally, 3-methylhistidine, a marker of inflammation^50^, was found to increase with time in all patients. However, its levels were lower in patients developing hypoxaemia at all time points, probably indicating its utilization by inflammatory cells.

Taken together, these findings suggest that patients progressing to hypoxaemia are more prone to produce an exaggerated stress response to CABG. This is in accordance with our previous findings regarding predictive biomarkers of lung injury based on samples collected at 16 h post-CPB^30^, indicating that the development of hypoxaemia can be predicted earlier than previously thought.

### Study limitations

Several practical limitations must be acknowledged. Although our study is the largest of its type published to date, sample sizes are still relatively small and involve comparisons between multiple groups. Since the study was performed on 50 consecutive patients undergoing CABG, we could not match for sex differences. Furthermore, we did not have a new set of samples to validate our results, and therefore, the validation of the identified metabolic biosignature for the progression into hypoxaemia requires further studies. We did not have serum samples on the preoperative day, and the second and third day postoperatively. Because the levels of metabolites such as phospholipids and amino acids are different in serum and plasma, direct comparison could not be achieved for some metabolites, and hence, we reported changes over time in percentages.

The present study is an attempt to elucidate the pathways of early progression to postoperative lung injury. It was our aim to improve understanding of the mechanistic underpinnings of lung injury, and to pave the way for future research.

To the best of our knowledge, this is the first metabonomics study demonstrating the link between intra- and postoperative time-dependent metabolite changes and the later development of postoperative hypoxaemia. We found a unique metabolic signature that clearly discriminated unaffected from hypoxaemic patients at least 48 hours before the clinical signs of oxygen impairments. Also, the results indicate that metabotyping patients’ journeys early, during or just after the end of surgery, may have potential impact in hospitals for the early diagnosis of postoperative lung injury, and for the monitoring of therapeutics targeting disease progression.

## Materials and Methods

### Patient population and sample collection

The study is registered at ClinicalTrials.gov (identifier: NCT02475694) and it is ethically approved by the Danish Health Authority Committee. After obtaining informed consent, fifty consecutive patients scheduled for elective CABG with the use of CPB at Aalborg University Hospital were included in this study. Inclusion criteria were adults above 18 years of age and on treatment with statins. Exclusion criteria were treatment with steroids or other immune suppressor therapies. All experiments were conducted according to relevant guidelines and regulations.

Patients underwent an overnight fast and standardized anaesthetic, surgical and perfusion management. Immediately after anaesthesia, a pulmonary artery (PA) catheter was inserted, and after sternotomy, a left atrium (LA) catheter. Paired blood samples were collected simultaneously at baseline (before CPB), right after weaning from CPB (0 hour), and at 2, 4, 8, and 20 hours after weaning from CPB (when the catheters were removed). Three patients were unable to provide samples at 20 hours postoperatively since their LA catheters were displaced. A total of 594 blood samples were collected and serum was obtained through standard hospital protocols. In addition, three patients were unable to provide samples the second and third day postoperatively, hence, a total of 144 blood samples were drawn from the radial artery the day before surgery, and at 48 and 72 hours after weaning from CPB, and plasma was obtained. Both serum and plasma aliquots were stored at −80 °C until analysis.

Postoperatively, patients were treated with supplementary oxygen to achieve peripheral oxygen saturations above 95%. In order to standardize the measurements, arterial blood samples were taken while patients had been spontaneously breathing atmospheric air for 10 minutes.

### Sample preparation

Before NMR analysis, samples were thawed for 30 min at 4 °C, vortexed, and subsequently centrifuged for 5 min at 12100 g and 4 °C. A total of 400 μL of the clear supernatant was mixed with 200 μL 0.20 M phosphate buffer (pH 7.4, 99% ^2^H_2_O, 0.30 mM DSA-d_6_ (1,1,2,2,3,3-hexadeutero-4, 4-dimethyl-4-silapentane-1-ammonium trifluoroacetate)) in a 5 mm NMR tube. The pH was 7.4 ± 0.04. During the whole process samples were kept on ice.

### NMR experiments

^1^H NMR spectra were recorded on a BRUKER AVIII-600 MHz NMR spectrometer (BrukerBioSpin, Rheinstetten, Germany) equipped with a cryogenically cooled, triple-resonance CPP-TCI probe, at a temperature of 298.1 K (25 °C). Spectral acquisition was controlled using the TopSpin 3.1 software (Bruker BioSpin).

T_2_ filtered Carr-Purcell-Meiboom-Gill (CPMG)^51^ experiments with water presaturation were acquired with the following parameters: 65536 data points over a spectral width of 20 ppm; 256 scans for serum and 128 scans for plasma samples; 32 dummy scans; a fixed receiver gain (RG) of 203; and a relaxation delay (D1) of 4 s, during which presaturation of the water resonance was achieved by continuous irradiation at γB_1_/2π = 25 Hz. T_2_ filtering was achieved with a repeated τ-180°-τ pulse sandwich with τ = 300 μs, repeated 256 times for serum samples and 128 times for plasma samples for a total of 80 and 40 ms, respectively.

To achieve more in-depth information about sample lipoprofiles, a one-dimensional diffusion-edited pulse sequence was used, with the following parameters: 65536 data points; 30 ppm spectral width; 128 scans; RG = 114; and D1 = 4 s, during which, presaturation of the water resonance was achieved by continuous irradiation at γB_1_/2π = 25 Hz. Diffusion filtering was achieved by inserting a stimulated-echo element into the pulse sequence between excitation and detection (BRUKER standard pulse program ledbpgppr2s1d). The diffusion time Δ, during which water presaturation was effective, was 120 ms, and bipolar sine-shaped gradients of 52.5 G/cm and 1.5 ms length were used (δ = 3 ms) for diffusion encoding. An Eddy-current delay of 5 ms before acquisition was used.

### Data processing

Spectral processing was carried out in TopSpin 3.1. FIDs were exponentially multiplied, corresponding to a line broadening of 0.3 Hz (CPMG) and 1 Hz (diffusion-edited), Fourier transformed, phase and baseline corrected, and calibrated (to the chemical shift of the methyl signal of L-alanine at 1.48 ppm for CPMG spectra, and to the methyl signal of N-acetylglucosamine at 2.04 ppm for diffusion-edited spectra). Spectra were reduced to buckets of 0.001 ppm width, and the water region between 4.65 and 4.95 ppm was excluded, using AMIX software (Analysis of MIXtures, v.3.9.10, Bruker BioSpin, Germany).

Data was then exported to MATLAB R2011b, MathWork. The binned data was generalized log transformed^52^ to enhance small signals in the spectrum, normalized to either the DSA-d_6_ peak intensity (CPMG) or to total intensity (diffusion-edited), and mean centred.

### Multivariate data analysis

Data analysis was performed in Matlab R2011b and SPSS (IBM^®^ Statistics v.22).

For multivariate analysis both unsupervised PCA and PLS and PLS-DA were applied using the PLS-Toolbox 6.5 (Eigenvector Research, Wenatchee, WA). PCA was applied, to find the main source of variation within the data, to check population homogeneity, and to identify outliers based on samples’ metabolic similarities and dissimilarities. PLS regression was applied to establish early metabolome associations with later outcome. NMR data were regressed to PaO_2_ values measured 72 hours postoperatively. In order to find spectral regions that correlating significantly with later PaO_2_ values, the reverse interval-PLS (riPLS) approach was applied. Regions found to correlate with PaO_2_ were then used to classify patients according to their diagnosis. For each classification model, a ROC curve, and a sensitivity and specificity were obtained. This information was used to evaluate the metabolome’s ability to predict later hypoxaemia.

For supervised modelling, a ten-fold Venetian-Blinds CV was employed. This validation involves: omitting 10 out of 100 samples from model development; developing parallel models from the reduced data; predicting the omitted samples; and comparing the predicted and actual values to provide an estimate of the model’s overall predictive power. For the PLS regression models, the overall predictive power was assessed by the cross-validated root mean square error (RMSECV) obtained from predicting PaO_2_ values. For PLSD-DA, the CV sensitivity and specificity values were used. To ensure that no random model performed equally well, or better, than the main PLS and PLS-DA models, permutation testing was also performed. Here we scrambled the PaO_2_ values and group labels (hypoxaemia/no-hypoxaemia) 500 times and subsequently performed multivariate modelling. The ‘true’ optimal PLS and PLS-DA models were then compared to the distribution of the permuted models, and significances were calculated using Wilcoxon’s sign rank test. A p-value < 0.004 was considered to be significant.

Models were visualized using scores and loadings plots. Each score represents a sample, while each loading represents the variation in a specific spectral region. Thus, the sum of all loadings determines the molecular signature or metabolic ‘fingerprints’ of a patient. The orientation of each loadings variable describes the up- and down-regulation of the corresponding bucket containing metabolite information. Spectral regions contributing to sample clustering were identified and quantified.

### Metabolite identification

For the identification process, ^1^H shifts and their corresponding ^13^C signals were analysed by running several 2D ^1^H-^1^H total correlation spectroscopy (TOCSY) and ^1^H-^13^C heteronuclear single-quantum correlation (HSQC) spectra. These signals were matched to The Human Metabolome Database^40^, Bruker BBIOrefcode Database (v. 2.7.0), and literature^30^,^53^,^54^.

For metabolite quantification, the NMR peaks were integrated by using the line shape analysis option in the AMIX Multi Integration tool.

### Further analysis and data representation

Metabolites and PaO_2_ levels are presented as mean ± standard deviation (SD) and percent change in tables, and as bar-plots. Percentage changes were calculated by the formula: (Y − X)/X∙100; where Y represents a sample collected at a time point different from its corresponding baseline sample, and X represents the baseline sample (pre-CPB for serum LA and PA samples; the day before surgery for plasma samples collected from the radial artery).

Several comparisons were undertaken on the data. First, we compared serum metabolic profiles recorded at six different time points (before CPB, 0, 2, 4, 8, and 20 hours post-CPB). Second, the metabolic profiles from before and right after weaning from CPB (0 hour) were compared in both PA and LA samples. Third, the impact of the duration of the surgical procedure on the human metabolome was evaluated. Fourth, patients’ metabolic journeys, from the day before surgery to the third postoperative day were analysed. Finally, the metabolome’s ability to predict hypoxaemia at an early stage was assessed, and the metabolites that were associated with the development of later lung injury were quantified and graphed.

Because patients received nutritional support on days two and three, and since plasma and serum matrices are biologically different in terms of the levels of several amino acid and phospholipid^55^,^56^,^57^, we chose to differentiate between plasma and serum results, and to show results as percent changes. Hence, only the significant results obtained from plasma samples are presented in the result section, while additional results are presented in the supplementary part.

Differences between hypoxaemic patients and unaffected patients were evaluated by the χ^2^ test for discrete clinical variables, and by the t-test for continuous variables. Differences in paired LA and PA intensities of each metabolite, and differences in paired samples collected before commencement of CPB and just after weaning from CPB (0 hour), were evaluated with the paired t-test or Wilcoxon signed-rank test, depending on the fulfilment of the normality assumptions. The interactions between time-, disease-, and LA/PA-dependent metabolic changes were determined by factorial ANOVA, with Tukey’s post-hoc test for multiple comparisons.

Tests of correlation were performed by calculating the Pearson correlation coefficient (*r*_*p*_). The 2-sided Fisher exact test was used to determine differences in frequency distributions. Statistical significance was defined as a p-value ≤ 0.05.

## Additional Information

**How to cite this article**: Maltesen, R. G. *et al*. Metabotyping Patients’ Journeys Reveals Early Predisposition to Lung Injury after Cardiac Surgery. *Sci. Rep.* **7**, 40275; doi: 10.1038/srep40275 (2017).

**Publisher's note:** Springer Nature remains neutral with regard to jurisdictional claims in published maps and institutional affiliations.

## Supplementary Material

Supplementary Material

## Figures and Tables

**Table 1:** Clinical and procedural characteristics of the study population.

	All (n = 50)	Unaffected (n = 18)	Mild Hypoxaemia (n = 23)	Severe Hypoxaemia (n = 9)	p-value* (2-sided)
**Subject characteristics**					
Age, mean ± SD, years	65.8 ± 9.7	66.6 ± 8.4	65.6 ± 10.7	64.8 ± 10.6	0.9
Male, n (%)	41 (82)	18 (100)	18 (78)	5 (56)	0.01
BMI, mean ± SD, kg/m^2^	27.3 ± 4.0	27.8 ± 3.0	26.9 ± 4.5	27.6 ± 4.6	0.8
Diabetes mellitus, n (%)	14 (28)	4 (22)	6 (26)	3(33)	0.1
Smokers (yes/no/unknown)	16/30/4	5/11/2	6/15/2	5/4/0	0.4
COPD, n (%)	7 (14)	3 (17)	2 (9)	2 (22)	0.6
**Cardiothoracic Intensive Care**, **mean** ± **SD**					
PaO_2_/FiO_2_ before extubation, kPa	38.2 ± 8.7	38.7 ± 6.4	40.0 ± 8.1	32.7 ± 12.4	0.09
Length of mechanical ventilation, h	7.1 ± 4.1	6.9 ± 4.4	7.2 ± 4.2	7.2 ± 3.4	0.9
Length of stay, h	23 ± 6	21.9 ± 1.2	22.8 ± 5.5	26.3 ± 10.3	0.2
Readmission	2	0	0	2	0.5
**Surgical procedure**, **mean** ± **SD**					
Surgery, min	196 ± 50	177 ± 38	205 ± 46	211 ± 69	0.12
Time on CPB, min	64 ± 29	57 ± 21	65 ± 26	73 ± 47	0.4
Cross clamp, min	33 ± 19	25 ± 11	36 ± 19	39 ± 26	0.09
Gain volume, L	2.0 ± 0.9	1.9 ± 1.1	2.1 ± 0.8	1.8 ± 0.9	0.6
Cardioplegia solution, mL	437 ± 118	413 ± 98	447 ± 115	458 ± 164	0.6
**72 hours post-CPB**, **mean** ± **SD**					
PaO_2_, kPa	7.8 ± 1.4	9.2 ± 0.5	7.5 ± 0.5	5.6 ± 0.4	<0.0001
PaO_2_/FiO_2_, kPa	37.0 ± 6.6	43.9 ± 2.5	35.7 ± 2.3	26.5 ± 2.1	<0.0001
Weight gain, kg	4.0 ± 3.1	2.7 ± 2.0	4.2 ± 3.6	6.0 ± 2.4	0.03
C-reactive protein, mg/L	146 ± 66	111 ± 36	152 ± 70	196 ± 73	0.008
Leukocytes (n × 10^9^)	9.9 ± 3.2	8.5 ± 2.6	10.4 ± 3.2	11.8 ± 3.3	0.03
Pneumonia¤	2	0	0	2	0.5
Atrial fibrillation	15	4	9	2	0.4
Length of hospital stay, days#	8 ± 4	8 ± 5	8 ± 2	11 ± 6	0.09

^¤^Clinical judgment leading to extended antibiotic treatment.

^#^Stay at the University Hospital.

^*^Analysis of variance (ANOVA) and chi-squared tests were used to calculate the significance between groups. Annotations: n, number of subject; SD, standard deviation; BMI, body mass index; COPD, chronic obstructive pulmonary disease; CPB, cardiopulmonary bypass; PaO_2_, partial pressure of oxygen in arterial blood; FiO_2,_ fraction of inspired oxygen (21%); Surgery, from skin incision to last suture.

**Table 2:** Metabolite changes as a consequence of CPB.

Metabolites	Pre-CPB						Post-CPB	
	PA (μmol/L)		LA (μmol/L)		PA		LA	
	Mean	SD	Mean	SD	Change(%)	p-value	Change(%)	p-value
Inosine	10.2	2.5	10.1	2.6	−18	<0.0001	−14.4	0.003
Hypoxanthine	13.9	1.6	13.7	1.9	−21.1	<0.0001	−20.3	<0.0001
Tryptophan	140.4	27.6	140.2	26.8	−22.2	<0.0001	−24.7	<0.0001
1-Metylhistidine	80.8	13.1	79	14.2	−10.1	0.001	−7.4	0.03
Uric acid	50	9.1	49.1	8.5	−7.9	0.03	−6.2	0.08
Xanthine	96.6	12.6	95.8	12.9	−16.2	<0.0001	−15.7	<0.0001
MUFA	786.3	213.3	771.9	202.2	−25.1	<0.0001	−24.5	<0.0001
Diacylglycerol	43.6	8.8	44.5	10.4	12.7	0.003	14.6	0.003
Phosphoenolpyruvate	174.5	54.1	174.2	50.1	7.5	0.10	10.3	0.06
N-acetyl‐galactosamine	209.8	85.3	205.9	98.8	21.3	0.02	15.8	0.10
β-Glucose	5043.2	1616	5015	1554.1	39.5	<0.0001	38	<0.0001
α-Glucose	2144.8	643	2127.3	619.9	35.9	<0.0001	36.1	<0.0001
4.60 ppm*	141.9	42.6	134.7	37.8	−24.9	<0.0001	−27.2	<0.0001
DAGPL	222.3	48.9	217.3	47.5	−19.1	<0.0001	−25	<0.0001
Phosphatidylcholine	531.8	73.9	520.4	75.3	−16.6	<0.0001	−17.7	<0.0001
GPC	137.4	15.6	134.9	15.8	−7.1	0.001	−7.4	0.001
Lactate	1495.3	585.7	1466.7	557.7	32.8	<0.0001	35.8	<0.0001
Glycerol	4870	1820	4763.5	1801	10	0.08	11.3	0.06
Choline& Sphingomyelin	793.4	140.3	788.6	132.9	−17.5	<0.0001	−17.4	<0.0001
Creatine	104.5	15.8	105.8	16.3	32.8	<0.0001	31.4	<0.0001
PUFA	543.5	145.3	535.5	140.1	−26.8	<0.0001	−26.7	<0.0001
Citrate	101.7	13.9	100.9	12.9	30.2	<0.0001	27.8	<0.0001
Pyruvate	43.6	16.7	41.9	16.5	61.3	<0.0001	67.3	<0.0001
3-Hydroxybutyric acid	168.3	45.9	167.1	45.7	−17.4	0.001	−17.2	0.001
Acetoacetate	120	50.4	120	50.8	−22.6	0.004	−23.1	0.003
Acetate	100.3	42.5	98.9	36.8	−19.9	0.060	−18	0.08
Lysine	489.2	45.9	484.8	47.5	−3.3	0.10	−3.2	0.12
Arginine	337.4	38.1	334.5	38.4	−6.1	0.009	−5.9	<0.0001
Free fatty acid	515.2	199.4	494.9	175.8	−30.2	<0.0001	−29.4	<0.0001
Alanine	482.8	92.9	475.7	88.8	15.1	<0.0001	15.8	<0.0001
Ethanol	492.1	199.3	490.8	223.4	−46.3	<0.0001	−45.2	<0.0001
Isobutyrate & Carnitine	32.1	4.7	31.6	5.2	−7.4	0.01	−6.8	0.03
Isoleucine	116.8	17.3	116.2	18.4	−14.4	<0.0001	−14.2	<0.0001
Leucine	275.3	37	272.5	38.2	−7.5	0.008	−6.9	0.02
Lipoproteins	3575.7	852.6	3512.4	812.3	−30.4	<0.0001	−30.2	<0.0001
Cholesterol	50.6	12.4	50.2	11	−34.5	<0.0001	−32.2	<0.0001

Metabolite levels found in pulmonary artery (PA) and left atrial (LA) samples collected before and after cardiopulmonary bypass (CPB). Mean change in percent was calculated using the formula: (post-CPB − pre-CPB)/pre-CPB * 100. Significance was assessed by the paired t-test or Wilcoxon signed-rank test, depending on the distribution of the data. Abbreviations: MUFA, mono-unsaturated fatty acids; DAGPL, diacylglycerophosphocholine; GPC, glycerophosphocholine; 4.60 ppm*, unassigned metabolite.

**Table 3:** The chronological metabolic events for the progression to postoperative lung injury.

Metabolites	Pre-CPB	Post-CPB (0 hour)	2 hours	4 hours	8 hours	20 hours
Trigonelline	√	√	√	√	√	√
3-Methylhistidine	√	√	√	√	√	√
Glycine	√	√	√	√	√	√
Inosine	√	√	√	√	√	√
Tyrosine	√	√	√	√	√	√
Dopa	√	√	√	√	√	√
Isobutyrylglycine	√	√	√	√	√	√
Malonate	√	√	√	√	√	√
Monounsaturated fatty acids (MUFA)		√		√	√	√
Free fatty acids				√	√	√
Polyunsaturated fatty acid (PUFA)		√	√	√	√	√
Glycerol		√	√			
Lipoproteins		√	√	√	√	√
Cholesterol		√		√	√	√
Phosphatidylcholine		√	√	√	√	
Choline		√	√	√		
Hypoxanthine		√	√	√	√	√
Xanthine		√	√	√	√	√
1-Methylhistidine		√	√			
Uric acid		√	√	√	√	√
Isoleucine		√	√			√
Leucine		√		√	√	√
Glutamate		√	√			
Alanine		√	√	√	√	√
Pyruvate		√	√	√	√	√
Citrate		√	√	√	√	√
N-Acetyl-Galactosamine (N-Ac-Gal)		√	√			√
3-Hydroxybutyric acid (3-HBA)		√		√	√	
Acetoacetic acid		√		√	√	
Acetate			√	√	√	√
Acetone			√	√	√	√
Triacylglycerol (TAG)				√	√	√
N-Acetyl-Glucosamine (N-Ac-Glc)						√

Annotation: ‘√’, the time point at which a metabolite was found to differ between patients.
