# Supplementary material for: Metabotyping Patients’ Journeys Reveals Early Predisposition to Lung Injury after Cardiac Surgery
Source: Sci Rep. 2017 Jan 11;7:40275. doi: 10.1038/srep40275 (PMC5225494; doi:10.1038/srep40275)
Supplement: Supplementary Material [file srep40275-s1.pdf]

Supplementary data

for article "Metabotyping Patients' Journeys Reveals Early Predisposition to Lung Injury after Cardiac Surgery"

Raluca Georgiana Maltesen, Bodil Steen Rasmussen, Shona Pedersen, Munsoor Ali Hanifa, Sergey Kucheryavskiy, Søren Risom Kristensen, Reinhard Wimmer

Table S1 A prolonged surgical procedure affects the metabolome. *Association between metabolome and duration of surgical procedure assessed by partial least square (PLS) regression. Both the calibrated (Cal) and cross-validated (CV) results are provided. At 4 hours post-CPB the association was weaker, suggesting that the metabolome had begun normalizing after prolonged surgical stress. No significant CV association was observed at 8 and 20 hourspostoperatively. Models with low predicted association are marked in grey.*

	CABG (196 ± 50 min.)		CPB (64 ± 29 min.)		Ischemia (33±19 min.)	
	R^2 Cal	R^2 CV	R^2 Cal	R^2 CV	R^2 Cal	R^2 CV
0 hour	-	-	0.94	0.76	0.92	0.75
2 hours	0.9	0.75	0.87	0.67	0.72	0.54
4 hours	0.82	0.6	0.7	0.41	0.7	0.44
8 hours	0.68	0.44	0.7	0.36	0.61	0.41
20 hours	0.74	0.39	0.55	0.32	0.63	0.38

Table S2 Metabolite time profiles in both left atrium (LA) and pulmonary artery (PA) samples. *Mean change in percentage was calculated using the formula: (post-CPB − pre-CPB)/pre-CPB*100. Significance was assessed by means of factorial ANOVA with Tukey's post hoc test for multiple comparisons. Abbreviations: TMAO, trimethylamine-N-oxide; N-Ac-Gal, N-acetyl-galactosamine; N-Ac-Glc, N-acetyl-glucosamine; FA, fatty acids; MUFA, monounsaturated fatty acids; PUFA, polyunsaturated fatty acids; DAG, diacylglycerol; PEP, phosphoenolpyruvate; 4.60ppm*, unassigned metabolite; DAGPL, diacylglycerophosphocholine; PC, phosphatidylcholine; GPC, glycerophosphocholine; SM, sphingomyelin.*

Metabolites	0 hour		2 hours		4 hours		8 hours		20 hours		p-value	
	**LA**	PA	**LA**	PA	**LA**	PA	**LA**	PA	**LA**	PA	**LA**	PA
**Trigonelline**	**-15**	-17	**-32**	-38	**-45**	-46	**-50**	-53	**-49**	-63	**0.010**	0.001
**Formate**	**2**	1	**-13**	-15	**-20**	-20	**-19**	-21	**-16**	-14	**<0.0001**	<0.0001
**Inosine**	**-14**	-18	**-27**	-25	**-28**	-29	**-24**	-25	**-24**	-30	**<0.0001**	<0.0001
**Hypoxanthine**	**-13**	-12	**-6**	-4	**-9**	-7	**4**	0	**12**	8	**<0.0001**	<0.0001
**Adenine**	**6**	12	**56**	45	**51**	47	**67**	67	**65**	54	**<0.0001**	<0.0001
**Tryptophan**	**-25**	-22	**-29**	-28	**-28**	-27	**-19**	-22	**-10**	-10	**<0.0001**	<0.0001
**Histidine**	**-1**	-1	**-9**	-11	**-14**	-15	**-13**	-14	**-14**	-16	**<0.0001**	<0.0001
**3-Metylhistidine**	**4**	4	**11**	9	**6**	6	**14**	15	**36**	32	**<0.0001**	<0.0001
**1- Metylhistidine**	**-7**	-10	**-7**	-11	**-12**	-14	**-5**	-9	**6**	0	**<0.0001**	<0.0001
**Phenylalanine**	**4**	4	**1**	-1	**1**	-1	**-4**	-6	**8**	6	**0.03**	0.02
**Uric acid**	**-6**	-8	**-13**	-17	**-13**	-16	**-2**	-6	**24**	17	**<0.0001**	<0.0001
**Tyrosine**	**4**	3	**-13**	-15	**-15**	-16	**-18**	-19	**-13**	-16	**<0.0001**	<0.0001
**Glucuronate**	**5**	6	**15**	13	**9**	9	**30**	26	**100**	94	**<0.0001**	<0.0001
**Xanthine**	**-16**	-16	**-26**	-26	**-24**	-25	**-14**	-16	**5**	3	**<0.0001**	<0.0001
**L-Dopa**	**0**	-1	**-8**	-10	**-11**	-12	**-11**	-12	**-7**	-9	**<0.0001**	<0.0001
**Fumarate**	**2**	1	**-11**	-13	**-9**	-9	**7**	-1	**8**	3	**0.04**	0.21
**Uridine**	**4**	3	**-5**	-7	**-9**	-9	**-14**	-15	**-23**	-28	**<0.0001**	<0.0001
**Urea**	**3**	1	**4**	1	**5**	4	**-4**	-6	**-24**	-27	**<0.0001**	<0.0001
**MUFA**	**-25**	-25	**49**	46	**77**	76	**76**	75	**63**	59	**<0.0001**	<0.0001
**TAG**	**-7**	-9	**123**	123	**159**	162	**146**	147	**136**	134	**<0.0001**	<0.0001

**N-Ac-Glc**	**5**	4	**17**	16	**26**	27	**41**	41	**60**	59	**<0.0001**	<0.0001
**DAG**	**15**	13	**6**	8	**4**	6	**12**	12	**19**	14	**0.001**	0.02
**PEP**	**10**	7	**22**	20	**30**	30	**16**	15	**-21**	-21	**<0.0001**	<0.0001
**N-Ac-Gal**	**16**	21	**15**	13	**13**	18	**-22**	-23	**-61**	-56	**<0.0001**	<0.0001
**β-Glucose**	**38**	39	**15**	14	**11**	12	**17**	17	**13**	13	**<0.0001**	<0.0001
**4.60 ppm***	**-27**	-25	**-24**	-27	**-18**	-20	**-8**	-12	**27**	31	**<0.0001**	<0.0001
**Malic acid**	**-7**	-3	**-31**	-33	**-39**	-39	**-39**	-39	**-41**	-37	**<0.0001**	<0.0001
**Ascorbate**	**2**	10	**-27**	-28	**-27**	-28	**-21**	-22	**-19**	-9	**<0.0001**	<0.0001
**DAGPL**	**-25**	-19	**-46**	-48	**-47**	-48	**-41**	-43	**-22**	-16	**<0.0001**	<0.0001
**PC**	**-18**	-17	**-3**	-6	**2**	1	**-1**	-3	**1**	1	**<0.0001**	<0.0001
**GPC**	**-7**	-7	**-14**	-16	**-14**	-17	**-14**	-16	**-10**	-11	**<0.0001**	<0.0001
**Lactate**	**36**	33	**5**	0	**6**	2	**-1**	-4	**-7**	-11	**<0.0001**	<0.0001
**Glycerol**	**11**	10	**26**	20	**34**	32	**1**	-1	**-45**	-46	**<0.0001**	<0.0001
**Glycine**	**-3**	-4	**-7**	-11	**-12**	-13	**-20**	-21	**-30**	-33	**<0.0001**	<0.0001
**α-Glucose**	**36**	36	**16**	14	**12**	13	**18**	18	**12**	11	**<0.0001**	<0.0001
**Taurine**	**-1**	-2	**-10**	-11	**-14**	-13	**-17**	-17	**-25**	-26	**<0.0001**	<0.0001
**Choline, SM**	**-17**	-18	**-9**	-10	**-3**	-4	**3**	2	**1**	1	**<0.0001**	<0.0001
**TMAO**	**-4**	-5	**-10**	-12	**-14**	-14	**-15**	-16	**-18**	-21	**0.03**	0.007
**Malonate**	**-1**	-2	**-4**	-6	**-7**	-6	**-8**	-7	**-7**	-8	**0.80**	0.81
**Creatinine**	**-2**	-2	**-3**	-3	**-4**	-4	**-4**	-4	**-5**	-6	**0.09**	0.04
**Creatine**	**31**	33	**29**	26	**19**	23	**15**	12	**8**	9	**<0.0001**	<0.0001
**Lipoic acid**	**-5**	-5	**-15**	-17	**-39**	-38	**-58**	-59	**-72**	-72	**<0.0001**	<0.0001
**PUFA**	**-27**	-27	**38**	35	**60**	59	**62**	61	**59**	58	**<0.0001**	<0.0001
**Citrate**	**28**	30	**27**	25	**41**	41	**22**	22	**2**	2	**0.01**	0.01
**Glutamine**	**-1**	-1	**-17**	-18	**-24**	-24	**-24**	-24	**-26**	-28	**<0.0001**	<0.0001
**Pyruvate**	**67**	61	**36**	29	**38**	35	**32**	28	**41**	33	**<0.0001**	<0.0001

**Glutamate**	**1**	0	**-2**	-3	**-4**	-5	**-7**	-7	**-5**	-6	**0.21**	0.15
**3-HBA**	**-17**	-17	**-19**	-20	**-14**	-15	**-5**	-6	**-13**	-14	**0.007**	0.003
**Acetoacetic acid**	**-23**	-23	**-8**	-8	**0**	1	**9**	8	**-25**	-26	**0.02**	0.02
**Acetone**	**5**	-1	**108**	96	**139**	132	**146**	134	**97**	87	**<0.0001**	<0.0001
**Acetate**	**-18**	-20	**-37**	-39	**-44**	-44	**-48**	-48	**-74**	-74	**<0.0001**	<0.0001
**Lysine**	**-3**	-3	**-9**	-11	**-18**	-19	**-23**	-24	**-30**	-32	**<0.0001**	<0.0001
**Proline**	**-3**	-3	**-10**	-11	**-18**	-18	**-22**	-23	**-24**	-26	**<0.0001**	<0.0001
**Arginine**	**-6**	-6	**-9**	-10	**-20**	-20	**-30**	-31	**-39**	-39	**<0.0001**	<0.0001
**Free FA**	**-29**	-30	**221**	206	**266**	260	**226**	219	**222**	208	**<0.0001**	<0.0001
**Alanine**	**16**	15	**-3**	-6	**-11**	-11	**-10**	-10	**-10**	-13	**<0.0001**	<0.0001
**Ethanol**	**-45**	-46	**-31**	-35	**-34**	-32	**-35**	-34	**-36**	-35	**<0.0001**	<0.0001
**Isobutyrylglycine**	**-12**	-13	**-29**	-30	**-42**	-42	**-55**	-55	**-70**	-71	**<0.0001**	<0.0001
**Isobutyrate & Carnitine**	**-7**	-7	**-9**	-11	**-16**	-18	**-31**	-32	**-41**	-42	**<0.0001**	<0.0001
**Valine**	**-3**	-3	**-9**	-11	**-18**	-17	**-25**	-26	**-33**	-35	**<0.0001**	<0.0001
**Isoleucine**	**-14**	-14	**-28**	-30	**-39**	-39	**-44**	-44	**-33**	-35	**<0.0001**	<0.0001
**Leucine**	**-7**	-8	**-5**	-7	**-8**	-9	**-11**	-13	**-11**	-14	**0.004**	0.000
**Lipoprotein**	**-30**	-30	**30**	28	**52**	52	**60**	58	**64**	60	**<0.0001**	<0.0001
**Total Cholesterol**	**-32**	-35	**1**	0	**20**	18	**33**	30	**38**	28	**<0.0001**	<0.0001

Table S3 Early prediction of hypoxaemia. *Partial least squares (PLS) regression and partial least squares –discriminant analysis (PLS-DA) results. The results show both the calibrated (Cal) and Venetian-Blinds cross-validated (CV) coefficient of association (R^2), specificity, sensitivity, and class error. Each model was built on 100 samples collected from 50 patients from the left atrium and pulmonary artery at one of the mentioned time points. For validation, the Venetian-Blinds cross-validation with 10 segments was applied. 10 consecutive samples were removed and a model was created. Subsequently, the 10 samples were predicted. This procedure was repeated until all samples were removed once. Permutation testing was applied for each model. A p-value ≤ 0.004 was considered significant.*

	PLS regression (Metabolome vs. PaO_2)					PLS-DA (Metabolome vs. Outcome (hypoxaemia: yes/no))						
	**R^2**		**RMSE**		**Permutation**	**Sensitivity**		**Specificity**		**Class error**		**Permutation**
	**Cal**	**CV**	**Cal**	**CV**	**p-value**	**Cal**	**CV**	**Cal**	**CV**	**Cal**	**CV**	**p-value**
**Pre-CPB**	0.94	0.707	0.33	0.75	0.004	0.83	0.78	0.95	0.84	0.11	0.19	0.004
**0 hour**	0.98	0.918	0.15	0.4	<0.001	1	0.88	0.98	0.92	0.01	0.1	<0.001
**2 hours**	0.98	0.934	0.13	0.36	<0.001	1	0.91	0.98	0.94	0.01	0.1	<0.001
**4 hours**	0.98	0.954	0.12	0.3	<0.001	0.97	0.88	0.92	0.88	0.06	0.12	<0.001
**8 hours**	0.98	0.941	0.13	0.34	<0.001	0.97	0.94	0.97	0.92	0.03	0.07	<0.001
**20 hours**	0.98	0.903	0.19	0.44	<0.001	0.96	0.93	0.97	0.91	0.04	0.08	<0.001
